# Xenon-Enhanced Dual-Energy CT Imaging in Combined Pulmonary Fibrosis and Emphysema

**DOI:** 10.1371/journal.pone.0170289

**Published:** 2017-01-20

**Authors:** Keishi Sugino, Masahiro Kobayashi, Yasuhiko Nakamura, Kyoko Gocho, Fumiaki Ishida, Kazutoshi Isobe, Nobuyuki Shiraga, Sakae Homma

**Affiliations:** 1 Department of Respiratory Medicine, Toho University Omori Medical Center, 6-11-1, Omori-nishi, Ota-ku, Tokyo, Japan; 2 Department of Diagnostic Radiology, Toho University Omori Medical Center, 6-11-1, Omori-nishi, Ota-ku, Tokyo, Japan; Universite de Bretagne Occidentale, FRANCE

## Abstract

**Background:**

Little has been reported on the feasibility of xenon-enhanced dual-energy computed tomography (Xe-DECT) in the visual and quantitative analysis of combined pulmonary fibrosis and emphysema (CPFE).

**Objectives:**

We compared CPFE with idiopathic pulmonary fibrosis (IPF) and chronic obstructive pulmonary disease (COPD), as well as correlation with parameters of pulmonary function tests (PFTs).

**Methods:**

Studied in 3 groups were 25 patients with CPFE, 25 with IPF without emphysema (IPF alone), 30 with COPD. Xe-DECT of the patients’ entire thorax was taken from apex to base after a patient’s single deep inspiration of 35% stable nonradioactive xenon. The differences in several parameters of PFTs and percentage of areas enhanced by xenon between 3 groups were compared and analyzed retrospectively.

**Results:**

The percentage of areas enhanced by xenon in both lungs were calculated as CPFE/IPF alone/COPD = 72.2 ± 15.1% / 82.2 ± 14.7% /45.2 ± 23.2%, respectively. In the entire patients, the percentage of areas enhanced by xenon showed significantly a positive correlation with FEV_1_/FVC (R = 0.558, P < 0.0001) and %FEV_1_, (R = 0.528, P < 0.0001) and a negative correlation with %RV (R = -0.594, P < 0.0001) and RV/TLC (R = -0.579, P < 0.0001). The percentage of areas enhanced by xenon in patients with CPFE showed significantly a negative correlation with RV/TLC (R = -0.529, P = 0.007). Xenon enhancement of CPFE indicated 3 different patterns such as upper predominant, diffuse, and multifocal defect. The percentage of areas enhanced by xenon in upper predominant defect pattern was significantly higher than that in diffuse defect and multifocal defect pattern among these 3 different patterns in CPFE.

**Conclusion:**

The percentage of areas enhanced by xenon demonstrated strong correlations with obstructive ventilation impairment. Therefore, we conclude that Xe-DECT may be useful for distinguishing emphysema lesion from fibrotic lesion in CPFE.

## Introduction

Xenon-enhanced dual-energy computed tomography (Xe-DECT) has recently been found to be feasible to assess visualizing lung ventilation [1–3]. This imaging technique has also been proven to be safe without serious side effects in both children and adults. In asthmatics or bronchiolitis obliterans setting, the ventilation defects seen on Xe-DECT showed significant correlations with the airflow obstruction on pulmonary function tests (PFTs) [4, 5]. Therefore, we believe that it will enable us to understand more precisely the distribution and localization of chronic obstructive pulmonary disease (COPD) including emphysematous lesions or combined pulmonary fibrosis and emphysema (CPFE).

CPFE has been proposed as an important phenotype of pulmonary fibrosis, defined by the presence of emphysema in the upper lobes and fibrosis in the lower lobes predominant on chest high-resolution computed tomography (HRCT) [6]. In patients with CPFE, it is difficult to ascertain whether subpleural cystic changes in the areas of fibrosis in the lower lobes are due to emphysema, honeycombing, bronchioloectasis, or a combination of these entities on chest HRCT.

There has been no comparative study yet of the patients with idiopathic pulmonary fibrosis (IPF alone), COPD and CPFE to visualize and quantify regional distributions of emphysema and fibrotic lesions based on the Xe-DECT data obtained by using three-material decomposition technique. Herein, this paper introduces the results of analyses undertaken to assess the feasibility of Xe-DECT in the visual and quantitative analysis of CPFE (IPF associated with emphysema) compared with IPF alone and COPD, as well as correlation with parameters of PFTs.

## Patients and Methods

This study was approved by our institutional review board (Toho university school of medicine ethical committee, approval number; 23–28) and written informed consent for the study protocols was obtained from all patients. Our clinical trial was registered with http://www.umin.ac.jp/english/ (UMIN000012523). The protocol for this trial and supporting TREND Statement Checklist are available as supporting information; see S1 Protocol and S1 Checklist. The primary outcome was the percentage of areas enhanced by xenon, and secondary outcomes were the relationship between the percentage of areas enhanced by xenon and PFTs parameters.

### Patients

Patients were divided into 3 groups as COPD associated with emphysema, IPF alone, and CPFE (IPF associated with emphysema).

**Fig 1:**
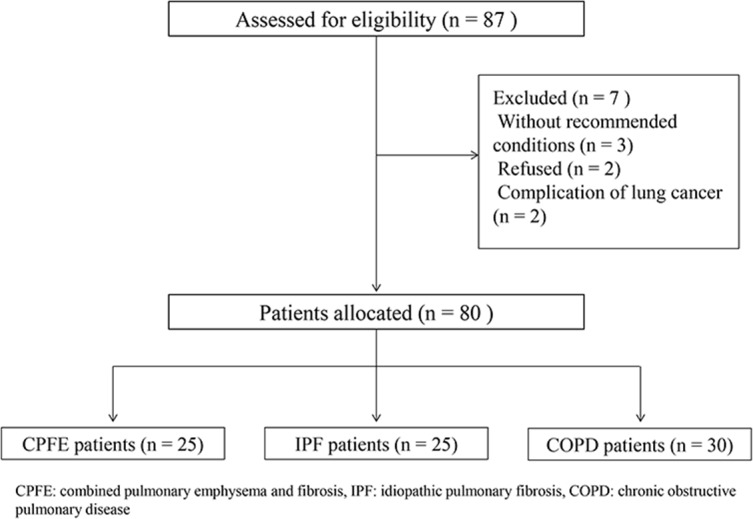
Study flow chart.

Thirty patients with COPD (27 males and 3 females, mean age: 73.1±9.0 years), 25 patients with IPF alone (18 males and 7 females, mean age: 74.4±6.6 years), and 25 patients with CPFE (20 males and 5 females, mean age: 74.1±6.4 years) who were diagnosed at our hospital were enrolled and reviewed retrospectively in our study (Fig 1). They underwent Xe-DECT and PFTs within a 1-month interval.

Exclusion criteria included patients with the following: unstable clinical condition, unable to maintain apnea for 15 second, and history of prior adverse reaction to xenon.

The diagnosis of IPF was made by a multidisciplinary clinic-radiological-pathological review of the patient data in accordance with the 2011 American Thoracic Society /European Respiratory Society/Japanese Respiratory Society/Latin American Thoracic Association (ATS/ERS/JRS/ALAT) [7].

The spirometrics criteria to define COPD were as follows; a FEV_1_/FVC ratio < 70% after the use of bronchodilators. The diagnosis of emphysema was based on focal areas or regions of low attenuation areas (LAAs) on chest HRCT [8]. The severity of COPD was evaluated based on the Global Initiative for Chronic Obstructive Lung Disease guidelines; grade 1 (%FEV_1_ predicted > 80%), grade 2 (50% < or = %FEV_1_ predicted < 80%), grade 3 (30% < or = %FEV_1_ predicted < 50%), and grade 4 (%FEV_1_ predicted < 30%) [9].

CPFE was regarded as IPF associated with emphysema which was defined as ≥ 10% emphysema on chest HRCT by modified criteria proposed by Ryerson, et al [10].

A consensus reading of chest CT images was analyzed independently by 2 pulmonologists (K.S., S.H.) and 2 radiologists (M.K, N.S.).

### Pulmonary function tests

All patients underwent spirometry and the measurement of diffusing capacity for carbon monoxide (DLco) by using a PFT system (Chestac-33, CHEST Co. Ltd., Tokyo, Japan). These PFTs were performed by 2 technicians according to the method described in the ATS criteria [11]. The DLco was measured by the single breath technique and adjusted for hemoglobin concentration.

Assessments of forced vital capacity (FVC), the forced expiratory volume in 1 second (FEV_1_), and the ratio of FEV_1_ to FVC (FEV_1%_) are based on the forced expiratory volume maneuver, in which the subject inhales maximally to total lung capacity (TLC) and then exhales forcefully and completely to residual volume (RV). The FEV_1_ is obtained from the volume-time spirogram by observing the volume exhaled in the 1 second of effort. Determination of FEV_1%_ provides the best index of airflow limitation. Moreover, the ratio of RV/TLC indicates air trapping, because RV increases to a greater extent than that seen in TLC in patients with obstructive lung diseases such as COPD. In contrast, DLco is a measure of the capacity to transfer gas from alveolar spaces into the alveolar capillary blood. The ratio of DLco to the alveolar volume (DLco/VA) implies that loss of lung volume secondary to mechanical abnormalities is accompanied by a parallel loss of diffusion capacity. The most common pattern in interstitial fibrosis such as IPF is for DLco to be reduced and DLco/VA to be slightly low or normal, because volume also is lost. Both DLco and DLco/VA are low with the loss of capillary surface area and blood volume in COPD with emphysema.

### Measurement of %LAA in COPD

The amount of emphysema, characterized by lung attenuation below -950 HU was measured by using automatical lung parenchymal instrument (syngo InSpace 4D, syngo InSpace Lung Parenchyma Evaluation).

### DECT image acquisition

All CT examinations were performed using a second generation dual source CT scanner (SOMATOM Definition Flash, Siemens Healthcare, Forchheim, Germany). First, an unenhanced single energy CT scan of the whole thorax of the patient was taken in caudio-cranial direction at 120 kV tube voltage, 150 mAs tube current time product, 64 x 0.6 mm collimation, 1.2 pitch and 0.5 s rotation time. Next the patients were fitted with face masks and elastic straps (King Systems, Nobelsville, IN, USA), and xenon dual energy CT scans of the patients’ entire thorax were taken from apex to base during breath hold after a single vital-capacity inspiration of 35% stable nonradioactive xenon with a Xenon gas re-breathing system (AZ-725, Anzai Medical, Tokyo, Japan). The scan parameters were as follows: 140kV with tin filter and 80kV tube voltages, 102 and 240 mAs (effective) tube current time product, a collimation of 64 x 0.6 mm, pitch of 0.55 and rotation time of 0.28 second. A medium sharp reconstruction kernel (D30f) was applied and reconstructed slice thickness was 2 mm at 1 mm interval.

Respiratory rate, oxygen saturation, and blood pressure were measured before and after the CT examinations. In addition, oxygen saturation was monitored throughout the entire study as well as tidal carbon dioxide and xenon concentrations monitored by using a sensor in the xenon gas inhalation system. All patients were asked to report any uncomfortable symptoms and troubles during the examination and were observed until 30 minutes after the CT examinations.

### Image post-processing

Xenon distribution maps were calculated by applying a 3-material decomposition algorithm of a commercial software package (syngo dual energy xenon and lung perfusion blood volume, Siemens Healthcare, Forchheim, Germany) at default parameter settings. For the calculation of the xenon and iodine maps the parameters were as follows: -1000 HU for air at 80 kV, -1000 HU for air at 140 kV, 60 HU for soft tissue at 80 kV, 54 HU for soft tissue at 140 kV, -1024 HU for minimum value, -500 HU for maximum value, and 4 for range.

Xenon maps were obtained as color coded images of the xenon distribution in the lung. Yellow areas indicated the presence of Xenon and suggested normal ventilation whereas brown or black areas indicated the partial or total absence of xenon and suggested ventilation defects. The maps could be also displayed in gray-scales or overlaid on non-xenon enhanced images.

### Image analysis -correlation of xenon ventilation and pulmonary function parameters-

Volumes of the whole lung as well as the right and left lung were calculated using a commercial software package (syngo volume, Siemens Healthcare, Forchheim, Germany). For the ventilation defects, volumes of ventilation defects were calculated by taking into account all pixels above 15 HU as optimal value because percentage of xenon ventilation defects significantly correlated with %LAA in COPD calculated by automatical lung parenchymal instrument (R = 0.803, P < 0.001). The correlation with several parameters of PFTs and percentage of areas enhanced by xenon between 3 groups were compared and analyzed.

All images were reviewed independently in random order by 2 radiologists with 30 and 8 years’ experience in chest radiology, who were blinded to the patients' history.

### Statistical analysis

All values are expressed as mean ± standard deviation. Statistical analysis for continuous values between 3 groups was performed using one-way analysis of variance (ANOVA) with Tukey’s multiple comparison or Kruskal-Wallis test with Steel-Dwass’s multiple comparison according to the presence or absence of normal distribution for 3 groups. When categorical variables were compared, a test of proportion difference followed by Bonferroni's multiple comparison was used. Pearson correlation coefficients were used to examine the correlation. Correlation coefficient values of ±0.4–1.0 were considered to indicate correlation [12]. Values of p < 0.05 were considered significant. Data analyses were performed using statistical software (JMP, version 10.0.0, SAS Institute, Cary, NC, USA).

## Results

### Baseline characteristics of patients

There were no significant differences between patients with CPFE and COPD in gender, age, height, weight, smoking history, smoking index value, and subtypes of emphysema on chest CT images. On the other hand, smoking history and smoking index values were significantly higher in patients with CPFE than in those with IPF alone. Baseline values of %FEV_1_ / FVC and %FEV_1_ in CPFE patients were significantly higher than those in COPD patients, whereas %TLC, %RV, and %DLco were significantly lower for CPFE. Patients with CPFE had increased %RV value and decreased %DLco/VA compared to those with IPF alone (Table 1).

**Table 1:** Baseline clinical characteristics of the study population.

Variable	CPFE	IPF alone	COPD	*P* value^†^	*P* value^‡^	*P* value^§^
Patients n	25	25	30			
*Age, yrs	74.1 ± 6.4	74.4 ± 6.6	73.1 ± 9.0	0.889	0.985	0.799
***Sex, male/female	20/5	18/7	27/3	0.907	1.000	0.261
*Height (cm)	159.1 ± 9.2	160.1 ± 8.6	161.7 ± 7.6	0.505	0.915	0.764
*Weight (kg)	57.0 ± 10.9	59.1 ± 9.6	52.9 ± 10.7	0.312	0.760	0.077
**BSA (m^2^)	1.57 ± 0.18	1.61 ± 0.16	1.54 ± 0.17	0.578	0.717	0.196
***Smoking history (+/-)	23/2	19/6	30/0	0.421	0.342	0.015
**Smoking index^#^	1092 ± 590	599 ± 505	1323 ± 691	0.369	0.005	< 0.0001
*%FVC (%)	85.2 ± 27.5	76.8 ± 19.9	93.2 ± 19.7	0.397	0.387	0.024
**FEV_1_/FVC (%)	78.6 ± 13.3	84.3 ± 8.6	47.2 ± 15.2	< 0.0001	0.221	< 0.0001
*%FEV_1_ (%)	98.1 ± 31.6	94.4 ± 21.0	65.9 ± 28.0	< 0.0001	0.880	< 0.0001
*%TLC (%)	83.5 ± 18.5	74.7 ± 18.1	115.8 ± 16.8	< 0.0001	0.194	< 0.0001
**%RV (%)	93.3 ± 24.8	76.5 ± 18.9	163.5 ± 49.2	< 0.0001	0.018	< 0.0001
*RV/TLC (%)	39.2 ± 10.4	37.1 ± 5.3	47.4 ± 10.7	0.005	0.701	0.0003
**%DLco (%)	49.9 ± 19.1	54.6 ± 19.1	63.1 ± 19.2	0.048	0.759	0.380
*DLco/VA (%)	47.2 ± 18.1	69.6 ± 19.3	41.3 ± 18.2	0.532	0.002	< 0.0001

^#^Smoking index; number of cigarettes consumed per day multiplied by years of smoking. NA; not available.

^**†**^: CPFE vs. COPD.

^**‡**^: CPFE vs. IPF alone^.^

^§^: IPF vs. COPD.

*: one-way ANOVA followed by Tukey’s multiple comparison.

**: Kruskal-Wallis test followed by Steel-Dwass’s multiple comparison.

***: a test of proportion difference followed by Bonferroni's multiple comparison.

Data are presented as mean ± SD. CPFE: combined pulmonary fibrosis and emphysema, COPD: chronic obstructive pulmonary disease, IPF: idiopathic pulmonary fibrosis, CL: centrilobular, PS: paraseptal, FVC: forced vital capacity, FEV_1_: forced expiratory volume in 1 s, TLC: total lung capacity, RV: residual volume, DLco: diffusion capacity for carbon monoxide, DLco/VA: diffusion capacity divided by the alveolar volume (DLco/VA).

### Safety

Common adverse event due to xenon inhalation during the study was dizziness (n = 3). However, the severity grade of the adverse event was very mild and disappeared within 30 minutes in all patients, and all patients could achieve the study.

### Image analysis of ventilation defect assessments

**Fig 2:**
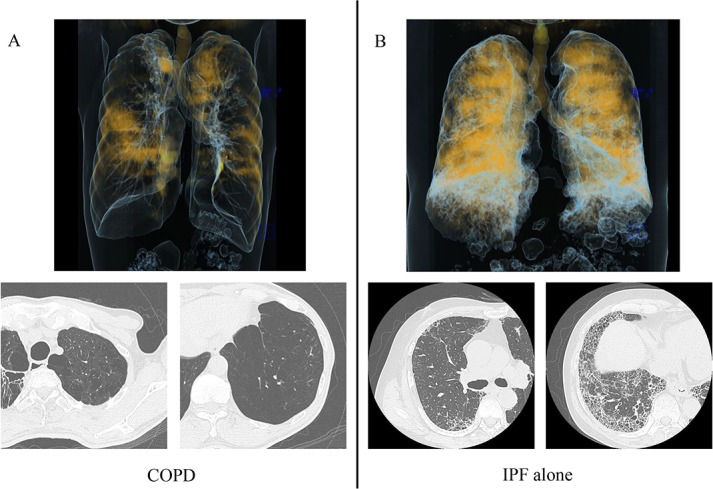
Image analysis of ventilation defect in COPD and IPF alone. (A) COPD shows largely focal xenon ventilation defects.(B) IPF alone shows slightly decreased xenon ventilation with several defects and volume loss with diffuse hypoventilation in the fibrotic lesion.

**Fig 3:**
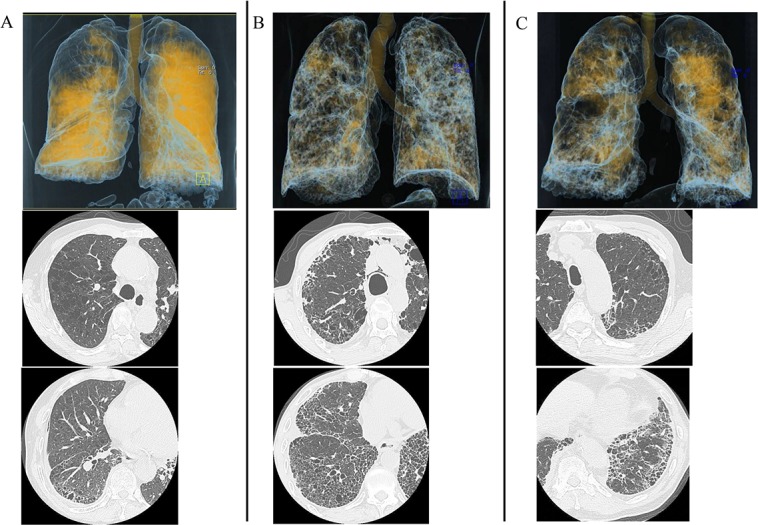
Image analysis of ventilation defect in CPFE. (A) CPFE of upper-predominant defect type shows xenon enhancement defect in emphysematous lesions affecting predominantly in both upper lobes, on the other hand relatively preserved in both lower lobes with sporadic fibrosis.(B) CPFE of diffuse defect type shows uneven diffuse defects of xenon enhancement in both lungs, without large enhancement defects like COPD.(C) CPFE of multifocal defect type shows multifocal defects of xenon enhancement in both lungs, without large enhancement defects like COPD.

**Fig 4:**
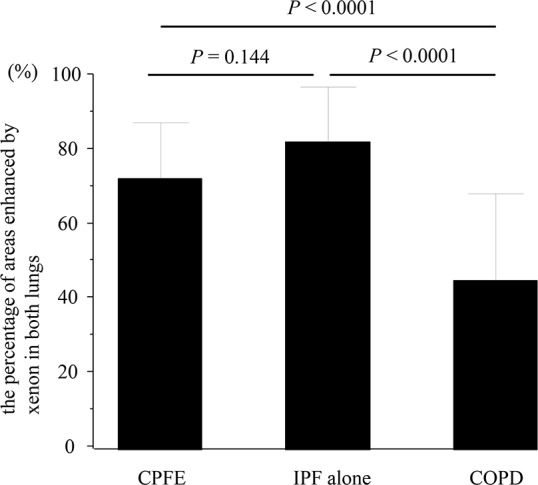
Comparison with the percentage of areas enhanced by xenon among CPFE, IPF alone, and COPD. The level of xenon enhancement in CPFE patients was significantly higher than that in COPD patients, but not significantly than IPF alone patients (CPFE vs. COPD; P < 0.0001, CPFE vs. IPF alone; P = 0.144, COPD vs. IPF alone; P < 0.0001: one-way ANOVA with Tukey’s correction for 3 comparison groups)

The distribution of xenon-enhanced areas in patients with COPD was focal with extensive areas of enhancement defects, whereas in patients with IPF alone it was relatively well preserved with slightly decreased xenon ventilation (Fig 2). On the other hand, xenon enhancement patterns in patients with CPFE were classified into the following 3 patterns according to xenon attenuation: i) upper lobe predominance defect pattern; xenon enhancement defect in emphysematous lesions affecting predominantly in both upper lobes, on the other hand relatively preserved in both lower lobes with sporadic fibrosis, ii) diffuse defect pattern; diffuse defects of xenon enhancement in both lungs without large enhancement defects such as COPD patients, iii) multifocal defect pattern; multifocal defects of xenon enhancement in both lungs with uneven xenon distribution (Fig 3). The percentage of areas enhanced by xenon in both lungs were calculated as CPFE/IPF alone/COPD = 72.2 ± 15.1% / 82.2 ± 14.7% /45.2 ± 23.2%, respectively. The level of xenon enhancement in CPFE patients was significantly higher than that in COPD patients, but not significantly than IPF alone patients (CPFE vs. COPD; P < 0.0001, CPFE vs. IPF alone; P = 0.144, COPD vs. IPF alone; P < 0.0001: one-way ANOVA with Tukey’s correction for 3 comparison groups) (Fig 4).

### Correlation of xenon ventilation and pulmonary function parameters

In the entire patients, the percentage of areas enhanced by xenon showed significantly a positive correlation with FEV_1_/FVC and %FEV_1_, and a negative correlation with %RV and RV/TLC (Table 2). Next, although there was no correlation between the percentage of xenon-enhanced areas and obstructive ventilation impairment such as FEV_1_/FVC, %FEV_1_, %RV, and RV/TLC in patients with IPF alone, there was significantly an inverse correlation between the percentage of xenon-enhanced areas and RV/TLC in patients with CPFE (Table 3).

**Table 2:** Correlation coefficients for relationships between the percentage of areas enhanced by Xenon and pulmonary function parameters in the whole patients (n = 80).

Variable	Correlation Coefficient	*P* value
%FVC (%)	0.137	0.253
FEV_1_/FVC (%)	0.558	< 0.0001
%FEV_1_ (%)	0.528	< 0.0001
%TLC (%)	-0.344	0.003
%RV (%)	-0.594	< 0.0001
RV/TLC (%)	-0.579	< 0.0001
%DLco (%)	0.033	0.788
DLco/VA (%)	0.297	0.032

FVC: forced vital capacity, FEV_1_: forced expiratory volume in 1 s, TLC: total lung capacity, RV: residual volume, DLco: diffusion capacity for carbon monoxide, DLco/VA: diffusion capacity divided by the alveolar volume (DLco/VA).

**Table 3:** Correlation coefficients for relationships between the percentage of areas enhanced by Xenon and pulmonary function parameters in CPFE patients (n = 25), IPF alone (n = 25), and COPD (n = 30).

	CPFE (n = 25)		IPF alone (n = 25)		COPD (n = 30)	
Variable	Correlation Coefficient	*P* value	Correlation Coefficient	*P* value	Correlation Coefficient	*P* value
%FVC (%)	0.284	0.169	0.358	0.118	0.521	0.003
FEV_1_/FVC (%)	-0.298	0.146	0.164	0.433	0.481	0.007
%FEV_1_ (%)	0.129	0.538	0.350	0.086	0.582	0.0007
%TLC (%)	0.048	0.819	0.345	0.092	0.194	0.303
%RV (%)	-0.375	0.064	0.299	0.147	-0.505	0.004
RV/TLC (%)	-0.529	0.007	0.087	0.680	-0.656	<0.0001
%DLco (%)	0.093	0.657	0.432	0.031	0.518	0.004
DLco/VA (%)	0.024	0.913	0.451	0.092	0.576	0.004

FVC: forced vital capacity, FEV_1_: forced expiratory volume in 1 s, TLC: total lung capacity, RV: residual volume, DLco: diffusion capacity for carbon monoxide, DLco/VA: diffusion capacity divided by the alveolar volume (DLco/VA).

#### Validation analysis of relationship between xenon defect pattern in CPFE and PFT findings

**Fig 5:**
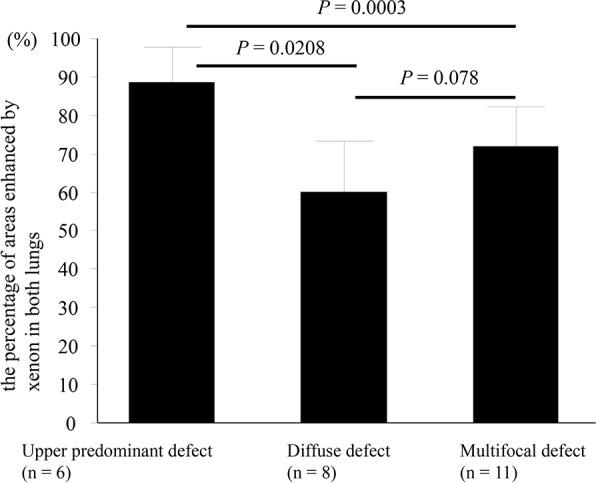
Comparison with the percentage of areas enhanced by xenon among 3 different patterns in CPFE. The percentage of areas enhanced by xenon in upper predominant defect pattern was significantly higher than that in diffuse defect (*P* = 0.0208) and multifocal defect pattern (*P* = 0.0003) among 3 different patterns in CPFE (one-way ANOVA with Tukeys correction for 3 comparison groups).

The percentage of areas enhanced by xenon in upper predominant defect pattern was significantly higher than that in diffuse defect (*P* = 0.0208) and multifocal defect pattern (*P* = 0.0003) among 3 different patterns in CPFE (one-way ANOVA with Tukeys correction for 3 comparison groups) (Fig 5). Diffuse defect pattern in CPFE showed restrictive ventilatory impairment predominant including severe decreased DLco and DLco/VA, whereas, upper predominant defect and multifocal defect pattern indicated obstructive ventilatory impairment predominant (Table 4).

**Table 4:** The relationship between pulmonary function parameters and 3 different patterns in CPFE.

Variable	Upper predominant defect type	Diffuse defect type	Multifocal defect type	*P* value^†^	*P* value^‡^	*P* value^§^
Patients n	6	8	11			
%FVC (%)	93.9 ± 29.7	61.8 ± 23.4	97.6 ± 18.4	0.043	0.008	0.945
FEV_1_/FVC (%)	78.1 ± 12.7	89.3 ± 8.6	71.1 ± 11.6	0.167	0.005	0.435
%FEV_1_ (%)	105.1 ± 34.4	75.9 ± 24.8	110.4 ± 28.1	0.168	0.042	0.928
%TLC (%)	84.4 ± 15.9	69.7 ± 16.1	93.0 ± 16.1	0.231	0.013	0.550
%RV (%)	80.4 ± 10.9	90.5 ± 23.5	102.3 ± 28.9	0.723	0.555	0.198
RV/TLC (%)	32.4 ± 8.2	46.4 ± 12.1	37.6 ± 7.1	0.026	0.122	0.517
%DLco (%)	50.9 ± 21.0	33.3 ± 11.6	61.6 ± 13.6	0.102	0.002	0.357
DLco/VA (%)	42.7 ± 24.9	36.4 ± 7.1	55.5 ± 14.9	0.789	0.087	0.312

NA; not available.

†: upper predominant defect type vs. diffuse defect type (one-way ANOVA with Tukey’s correction for 3 comparison groups).

‡: diffuse defect type vs. multifocal defect type (one-way ANOVA with Tukey’s correction for 3 comparison groups).

^§^: upper predominant defect type vs. multifocal defect type (one-way ANOVA with Tukey’s correction for 3 comparison groups).

Data are presented as mean ± SD.

FVC: forced vital capacity, FEV_1_: forced expiratory volume in 1 s, TLC: total lung capacity, RV: residual volume, DLco: diffusion capacity for carbon monoxide, DLco/VA: diffusion capacity divided by the alveolar volume (DLco/VA).

## Discussion

The percentage of areas enhanced by xenon showed strong correlations with obstructive ventilatory impairment. Xe enhancement of CPFE showed 3 distinct patterns in terms of Xe-DECT 3D images. This is the first study describing 3 distinct patterns of xenon enhancement and usefulness of Xe-DECT for distinguishing emphysema lesion from fibrotic lesion in CPFE.

Xe-DECT with a multiple-breath-hold technique has recently been found to be feasible to assess visualizing lung ventilation [4, 5]. More recently, Xe-DECT with a single-breath-hold technique could also depict pulmonary ventilation [13]. This Xe-DECT imaging technique appears to be safe without serious side effects in both children and adults since a xenon concentration of 35% is the approved maximum for clinical use. In the volunteers with normal lungs, xenon was distributed homogeneously throughout the entire lung [3, 13]. In asthmatics setting, the ventilation defects seen on Xe-DECT showed significant correlations with the airflow obstruction on pulmonary function tests (PFTs) and the airway wall thickening on chest CT [4]. Goo et al [5]. reported that volume percentages of hyperlucent lesions using CT densities and xenon-enhanced ventilation defects were correlated with PFT findings such as forced expiratory volume in 1 second (FEV_1_) and FEV_1_/forced vital capacity (FVC) in children with bronchiolitis obliterans. Park et al [14]. reported that ventilation patterns in patients with COPD were various and categorized into 4 patterns according to xenon attenuation (combination of hypo-, iso-, or hyperattenuating regions in the wash-in and wash-out periods) with the multiple-breath-hold technique. On the other hand, as described by Honda et al [13], areas of reduced ventilation such as bullae were always depicted as area of xenon-enhanced ventilation defects. Moreover, they reported that Xe-DECT with single-breath-technique demonstrated not only morphological findings of the whole lungs but also functional lung information [13]. Therefore, we believe that Xe-DECT with single-breath-technique is potentially of tremendous utility to help us depict three-dimensional structures and ventilation distribution of pulmonary parenchyma. Actually, in this study, the percentage of areas enhanced by xenon was calculated quantitatively and significant correlations between xenon-enhanced areas and parameters of PFT indicating obstructive ventilation impairment were observed.

A consensus definition of CPFE is not available at the moment. Although the term of CPFE was defined emphysema in the upper lobes and fibrosis in the lower lobes predominant on chest high-resolution computed tomography (HRCT) by Cottin et al [6], the most serious issue is that chest HRCT criterion to define CPFE lacks objectivity due to its vague nature. Patients with CPFE had a variety of extent of emphysema or pulmonary fibrosis. Thus, it is known that distinguishing from picture of emphysema and fibrosis is occasionally difficult because of great variety in different patterns of that distribution and proportion. Furthermore, it is difficult for CPFE patients to ascertain whether subpleural cystic changes in the areas of fibrosis are due to emphysema, honeycombing, bronchioloectasis, or a combination of these entities on chest HRCT. Therefore, we think that CPFE is by no means a simple concept, and this is why previous studies have led to a different outcome of patients with CPFE compared to those with IPF alone [15–17]. As a result, it is important for our clinicians to understand more precisely the distribution and localization of CPFE. There has been no comparative study yet of the patients with IPF alone, COPD or CPFE to visualize and quantify regional distributions of emphysema and fibrotic lesions based on the Xe-DECT data obtained by using three-material decomposition technique. Although distinguishing emphysema lesions from fibrotic lesions based on the Xe-DECT data obtained by using three-material decomposition technique was a new approach, it is quite possible that patients with CPFE who was diagnosed based on conventional criteria proposed by Cottin [6] and/or Ryerson [10] are mainly divided into 3 groups (upper predominant defect, diffuse defect, and multifocal defect pattern) according to differences of xenon enhancement pattern. As a result, it is interesting to note that some significant different pulmonary function impairments were found among these 3 groups. This study provides a first step in investigating differences of treatments and outcome among several phenotypes in CPFE. Thus, a single-breath technique may be applicable to Xe-DECT for obtaining pulmonary ventilation images. Also this technique would have the advantage of shortening the examination time and reducing the patient’s exposure to xenon gas.

There are several limitations in this study. First, this was a preliminary study from a single center with a relatively small sample size. Therefore, our results should be confirmed in a larger cohort. Second, recirculated xenon in blood flow can affect the influences on lung CT attenuation. In fact, xenon residence time in the lung is shorter with single-breath-hold technique than that with the multiple-breath-hold technique based on paper described by Hoag et al [18]. Third, the threshold of 15 HU for defining ventilation defect remain obscure because this threshold may differ with each CT machine and the conditions. Therefore, our data should be interpreted as preliminary.

In conclusion, the percentage of areas enhanced by xenon demonstrated strong correlations with obstructive ventilation impairment. As a result, this novel technique will be a promising tool to generate significant imaging analysis data, which may be useful for distinguishing emphysema lesion from fibrotic lesion in CPFE. As future prospects, we seek to design that a clinical study on whether follow-up Xe-DECT images of CPFE patients could be estimated on PFT findings.

## Supporting Information

S1 Checklist TREND Checklist. (PDF)

S1 Protocol (DOCX)

S1 File UMIN Clinical Trial. (PDF)
