# Supplementary material for: Xenon-Enhanced Dual-Energy CT Imaging in Combined Pulmonary Fibrosis and Emphysema
Source: PLoS One. 2017 Jan 20;12(1):e0170289. doi: 10.1371/journal.pone.0170289 (PMC5249235; doi:10.1371/journal.pone.0170289)
Supplement: S1 Protocol — (DOCX) [file pone.0170289.s002.docx]

**DECT image acquisition**

First, an unenhanced single energy CT scan of the whole thorax of the patient was taken in caudio-cranial direction at 120 kV tube voltage, 150 mAs tube current time product, 64 x 0.6 mm collimation, 1.2 pitch and 0.5 rotation time. Next the patients were fitted with face masks and elastic straps (King Systems, Nobelsville, IN, USA), and xenon dual energy CT scans of the patients’ entire thorax were taken from apex to base during breath hold after a single vital-capacity inspiration of 35% stable nonradioactive xenon with a Xenon gas re-breathing system (AZ-725, Anzai Medical, Tokyo, Japan). Finally a contrast enhanced dual energy CT scan was taken 18 seconds after the start of an intravenous injection of iodinated contrast material at a flow rate of 3.0 ml/s (total volume of 60-100 ml), followed by a 60 ml saline chaser bolus. The scan parameters were as follows: 140kV with tin filter and 80kV tube voltages, 102 and 240 mAs (effective) tube current time product, a collimation of 64 x 0.6 mm, pitch of 0.55 and rotation time of 0.28 second. A medium sharp reconstruction kernel (D30f) was applied and reconstructed slice thickness was 2 mm at 1 mm interval.

Respiratory rate, oxygen saturation, and blood pressure were measured before and after the CT examinations. In addition, oxygen saturation was monitored throughout the entire study as well as tidal carbon dioxide and xenon concentrations monitored by using a sensor in the xenon gas inhalation system. All patients were asked to report any uncomfortable symptoms and troubles during the examination and were observed until 30 minutes after the CT examinations.

**Image post-processing**

For the calculation of the xenon maps the parameters were as follows: -1000 HU for air at 80 kV, -1000 HU for air at 140 kV, 60 HU for soft tissue at 80 kV, 54 HU for soft tissue at 140 kV, -1024 HU for minimum value, -500 HU for maximum value, and 4 for range. For the calculation of the iodine maps the parameters were as follows: -1000 HU for air at 80 kV, -1000 HU for air at 140 kV, 60 HU for soft tissue at 80 kV, 54 HU for soft tissue at 140 kV, 1.95 for relative contrast material enhancement, -1024 HU for minimum value, -500 HU for maximum value, and 4 for range.

Xenon maps were obtained as color coded images of the xenon distribution in the lung. Yellow areas indicated the presence of Xenon and suggested normal ventilation whereas brown or black areas indicated the partial or total absence of xenon and suggested ventilation defects. The maps could be also displayed in gray-scales or overlaid on non-xenon enhanced images. Similarly, iodine maps were obtained as color-coded images of the iodine distribution in the lung with yellow-orange areas indicating the presence of iodine and suggesting normal perfusion of the lung and brown or black areas indicating the partial or total absence of iodine and suggesting perfusion defects.

**Statistical analysis**

All values are expressed as mean ± standard deviation. Statistical analysis for continuous values between 3 groups was performed using one-way analysis of variance (ANOVA) with Tukey’s multiple comparison or Kruskal-Wallis test with Steel-Dwass’s multiple comparison according to the presence or absence of normal distribution for 3 groups. When categorical variables were compared, a test of proportion difference followed by Bonferroni's multiple comparison was used. Pearson correlation coefficients were used to examine the correlation. Correlation coefficient values of ±0.4-1.0 were considered to indicate correlation [12]. Values of p < 0.05 were considered significant. Data analyses were performed using statistical software (JMP, version 10.0.0, SAS Institute, Cary, NC, USA).
