# Supplementary material for: Xenon-Enhanced Dual-Energy CT Imaging in Combined Pulmonary Fibrosis and Emphysema
Source: PLoS One. 2017 Jan 20;12(1):e0170289. doi: 10.1371/journal.pone.0170289 (PMC5249235; doi:10.1371/journal.pone.0170289)
Supplement: S1 File — (PDF) [file pone.0170289.s003.pdf]

UMIN-ICDR Clinical Trial

Name: keishi sugino **UMIN ID:** suginok-path

**Recruitment status**	Completed
**Unique ID issued by UMIN**	UMIN000012523
**Receipt No.**	R000014656
**Official scientific title of the study**	Xenon ventilation imaging using dual-energy computed tomography in combined pulmonary fibrosis and emphysema
**Date of disclosure of the study information**	2013/12/09
**Last modified on**	2016/06/04

* This page includes information on clinical trials registered in UMIN clinical trial registed system.
* We don't aim to advertise certain products or treatments

Basic information	
Official scientific title of the study	Xenon ventilation imaging using dual-energy computed tomography in combined pulmonary fibrosis and emphysema
Title of the study (Brief title)	Xe-DECT in CPFE
Region	Japan

Condition	
Condition	Emphysematous chronic obstructive pulmonary disease, Chronic fibrosing interstitial pneumonia, Combined pulmonary fibrosis and emphysema
Classification by specialty	Pneumology Radiology
Classification by malignancy	Others
Genomic information	NO

Objectives	
Narrative objectives1	The aim of this study was to assess the feasibility of xenon ventilation computed tomography (CT) in the visual and quantitative analysis of combined pulmonary fibrosis and emphysema (CPFE)
Basic objectives2	Safety,Efficacy
Basic objectives -Others	
Trial characteristics_1	Confirmatory
Trial characteristics_2	Pragmatic
Developmental phase	Not applicable

Assessment	
Primary outcomes	Efficacy and safety of Xe-DECT

Key secondary outcomes	

In outcomes field, the entry of just a few words such as "safety" or "efficiency" will not be accepted. Specify the name of outcome measures, including the time when you plan to measure. Usually, only one primary outcome is accepted. Write the other outcomes in "secondary outcomes" field.

Base	
Study type	Interventional

Study design	
Basic design	Parallel
Randomization	Non-randomized
Randomization unit	
Blinding	Open -no one is blinded
Control	Uncontrolled
Stratification	
Dynamic allocation	
Institution consideration	
Blocking	
Concealment	

Intervention	
No. of arms	3
Purpose of intervention	Diagnosis
Type of intervention	Device,equipment
Interventions/Control_1	the feasibility of xenon ventilation computed tomography (CT) in the visual and quantitative analysis of combined pulmonary fibrosis and emphysema (CPFE)
Interventions/Control_2	Comparison between CPFE and emphysematous COPD
Interventions/Control_3	Comparison between CPFE and chronic fibrosing interstitial pneumonia
Interventions/Control_4	
Interventions/Control_5	
Interventions/Control_6	
Interventions/Control_7	
Interventions/Control_8	
Interventions/Control_9	

Interventions/Control_10	

In interventions field, include the details of interventions, such as duration, amount, and frequency. If the intervention includes prescription or use of medical devices, duration is required.

Eligibility	
Age-lower limit	40 years-old <=
Age-upper limit	85 years-old >
Gender	Male and Female
Key inclusion criteria	1) Age of 40 <=, 85><br>2) Sex: male and female<br>3) Patients: hospital patinets and outpatinets<br>4) Accepted informed consents<br>5) Clinically suspected or confirmed chronic fibrosing interstitial pneumonia, emphysematous COPD, and CPFE
Key exclusion criteria	1) unstable clinical condition and complication of severe underlying diseases<br>2) unable to examine pulmonary function tests<br>3) history of prior reaction to iodinated contrast media<br>4) serum creatinine value >= 2mg/dl
Target sample size	100

Research contact person	
Name of lead principal investigator	Sakae Homma
Organization	Toho University Omori Medical Center
Division name	Department of Respiratory Medicine
Address	6-11-1, Omori-nishi, Ota-ku, Tokyo 143-8541, Japan
TEL	+81-3-3762-4151
Email	sahomma@med.toho-u.ac.jp

Public contact	
Name of contact person	Keishi Sugino
Organization	Toho University Omori Medical Center
Division name	Department of Respiratory Medicine
Address	6-11-1, Omori-nishi, Ota-ku, Tokyo 143-8541, Japan
TEL	+81-3-3762-4151
Homepage URL	
Email	ks142129_ikusou@ybb.ne.jp

Organization sending information	
Name of person sending information	Keishi Sugino
Organization	Toho University Omori Medical Center
Division name	Department of Respiratory Medicine
Address	6-11-1, Omori-nishi, Ota-ku, Tokyo 143-8541, Japan
TEL	+81-3-3762-4151

Email	ks142129_ikusou@ybb.ne.jp

Sponsor	
Institute	Toho University Omori Medical Center,Department of Respiratory Medicine
Institute	
Department	

Sponsor means an organization that is responsible for plan, deployment and report of the research including funding management. It doesn't mean funding agency". Therefore, all clinical trial should have the one.

Funding Source	
Organization	JSPS KAKENHI Grant
Organization	
Division	
Category of Funding Organization	
Nationality of Funding Organization	JAPAN

Other related organizations	
Co-sponsor	
Name of secondary funder(s)	

IRBs	
Research ethics review	YES
Post marketing survey by drug manufacture etc., specified by Japanese law.	Not applicable (Chose this category if you are not drug manufacture etc. in Japan)
Organization1	東邦大学医療センター大森病院呼吸器内科
Address1	東京都大田区大森西6-11-1
Tel1	+81-3-3762-4151
Email1	secret_respi@med.toho-u.ac.jp
Organization2	
Address2	
Tel2	
Email2	
Organization3	
Address3	
Tel3	
Email3	

Secondary IDs	

Secondary IDs	NO
Study ID_1	
Org. issuing International ID_1	
Study ID_2	
Org. issuing International ID_2	
IND to MHLW	

Institutions	
Institutions	

Other administrative information	
Date of disclosure of the study information	2013 Year 12 Month 09 Day

Progress	
Recruitment status	Completed
Date of protocol fixation	2012 Year 06 Month 09 Day
Anticipated trial start date	2012 Year 06 Month 10 Day
Last follow-up date	2014 Year 03 Month 31 Day
Date of closure to data entry	
Date trial data considered complete	2013 Year 03 Month 31 Day
Date analysis concluded	2013 Year 03 Month 31 Day

Related information	
URL releasing protocol	
Publication of results	Unpublished
URL releasing results	
Results	
Other related information	

UMIN user permited to amend	
UMIN ID1	
UMIN ID2	
UMIN ID3	

Management information	

Unique ID issued by UMIN	UMIN000012523
Status	Regist
Applicant for provisional registration	suginok-path(keishi sugino)
Date of provisional registration	2013 Year 12 Month 09 Day
Registrant	suginok-path(keishi sugino)
Registered date	2013 Year 12 Month 09 Day
Last modified by	suginok-path(keishi sugino)
Last modified on	2016 Year 06 Month 04 Day

Research Plan		
Registered date	Registrant	File name

Research case data specifications		
Registered date	Registrant	File name

Research case data		
Registered date	Registrant	File name

Back

For inquiries about the use of UMIN clinical trial registration system, use this contact form .Fort other inquiries, use this contact form

UMIN Infrastructure for Academic Activities
University hospital Medical Information Network
